# Effects of GSM and UMTS mobile telephony signals on neuron degeneration and blood-brain barrier permeation in the rat brain

**DOI:** 10.1038/s41598-017-15690-1

**Published:** 2017-11-14

**Authors:** Florence Poulletier de Gannes, Hiroshi Masuda, Bernard Billaudel, Emmanuelle Poque-Haro, Annabelle Hurtier, Philippe Lévêque, Gilles Ruffié, Murielle Taxile, Bernard Veyret, Isabelle Lagroye

**Affiliations:** 1 University of Bordeaux, IMS laboratory UMR-5218 CNRS, Talence, F-33405 France; 2 Kurume University School of Medicine, Department of Environmental Medicine, Kurume, Fukuoka J-830-0011 Japan; 3 University of Limoges, CNRS, XLIM, UMR 7252, Limoges, F-87000 France; 4 “Paris Sciences et Lettres” Research University / EPHE, Paris, F-75005 France

## Abstract

Blood-brain barrier (BBB) permeation and neuron degeneration were assessed in the rat brain following exposure to mobile communication radiofrequency (RF) signals (GSM-1800 and UMTS-1950). Two protocols were used: (i) single 2 h exposure, with rats sacrificed immediately, and 1 h, 1, 7, or 50 days later, and (ii) repeated exposures (2 h/day, 5 days/week, for 4 weeks) with the effects assessed immediately and 50 days after the end of exposure. The rats′ heads were exposed at brain-averaged specific absorption rates (BASAR) of 0.026, 0.26, 2.6, and 13 W/kg. No adverse impact in terms of BBB leakage or neuron degeneration was observed after single exposures or immediately after the end of repeated exposure, with the exception of a transient BBB leakage (UMTS, 0.26 W/kg). Fifty days after repeated exposure, the occurrence of degenerating neurons was unchanged on average. However, a significant increased albumin leakage was detected with both RF signals at 13 W/kg. In this work, the strongest, delayed effect was induced by GSM-1800 at 13 W/kg. Considering that 13 W/kg BASAR in the rat head is equivalent to 4 times as much in the human head, deleterious effects may occur following repeated human brain exposure above 50 W/kg.

## Introduction

Homeostasis of the brain microenvironment, essential for its normal function, is maintained by the blood-brain barrier (BBB), which consists of highly specialized endothelial cells, where tight junctions between adjacent cells restrict the paracellular diffusion of hydrophilic molecules. Several sources of stress, such as immobilization^1,2^, cold^3^, forced swimming^4^, ionizing radiation^5–7^, and heat^8^ have been reported to alter BBB permeability. Exposure to radiofrequency fields (RF) at high specific absorption rates (SAR expressed in W/kg) have also been reported to induce BBB leakage and are known to induce hyperthermia and heat stress. The use of RF-emitting mobile phones is subject to regulations and local RF exposure is limited to 2 W/kg to avoid heat stress due to RF absorption.

The literature reports contradictory data on the occurrence of BBB permeation following exposure to low-level RF fields, such as those emitted by mobile phones. Salford and colleagues^9–14^ consistently reported albumin extravasation over the BBB in the brains of rats exposed at whole-body SAR as low as 0.002 W/kg. The effect, dependent on whole-body SAR up to 0.2 W/kg, was observed up to 50 days after a single 2 h exposure. The presence of dark neurons, assumed to be degenerative, was also reported 50 days after a single 2 h exposure of rats to GSM signals (Global System for mobiles, i.e. 2 G, second generation). However, these authors did not rule out the possibility of experimental bias, leading to an overestimation of the number of dark neurons. More recently, another group detected albumin extravasation in the brains of male, but not female, rats, exposed to CW RF at 900 and 1800 MHz and very low brain-averaged SAR (BASAR) ranging from 0.0014 to 0.0043 W/kg^15^.

In contrast, the Hoffmann group^16^ previously reported that some BBB leakage occurred in the rat brain at a head-mainly SAR of 7.5 W/kg but not at lower SAR levels. Our group^17^ and several others found no significant BBB leakage in animals (mice and rats) exposed to RF.^18–28^ In these published reports, signal frequencies ranged from 898 to 2450 MHz, whole-body SAR from 0.25 to 4 W/kg, and BASAR from 0.3 to 6 W/kg. The animals in these studies were not only subjected to single exposures ranging from 45 min to 4 h, but also to repeated exposures, up to 90 min/day, for up to 104 weeks. Most of these studies were reviewed in 2010,^29^ as well as in a European report issued in 2015,^30^ and both concluded that there was no evidence that low-level RF exposure induced BBB leakage.

The aim of this work was to provide further evidence that mobile communication signals had no demonstrable effects on the permeability of the rat BBB or neuron degeneration. We extended our previously-published work^17^ to GSM-1800 and UMTS-1950 (Universal Mobile Telephone System, 3 G, third generation), with repeated exposures. A total of 1,120 rats was used in this project over three years. Two exposure regimens were applied: (i) single 2-h exposure with effects investigated immediately, one hour, and 1, 7, or 50 days later and (ii) repeated exposure (2 h per day, 5 days per week, for 4 weeks) with testing for effects immediately and 50 days later. BASAR levels tested were 0.026 and 0.26 W/kg (within the range of effective values in the Salford group studies), 2.6 W/kg, and 13.0 W/kg. In both approaches, cage-control and sham-exposed groups were included along with the four RF-exposed groups.

## Results

### Positive control

**Figure 1:**
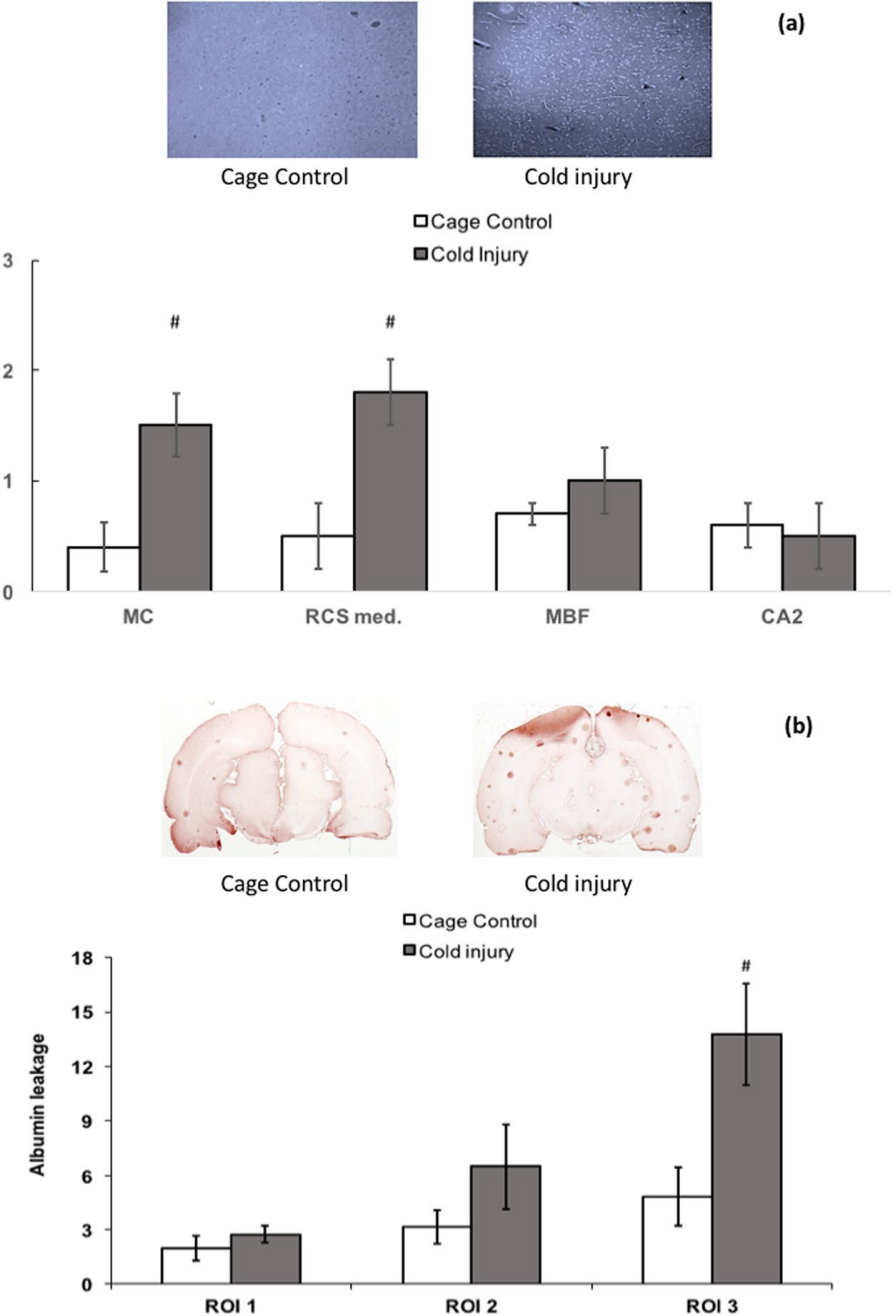
Degenerating neurons and endogenous albumin leakage after a cold injury (positive control). The rats were treated as indicated in the Methods. (**a**) Degenerating neurons identified using Fluoro-Jade B after a cold injury. Upper graphs show representative images of the presence of degenerating neurons in the motor cortex (MC) in cage control and positive control rats. Lower graph shows data of degenerating neurons in selected regions: motor cortex (MC), median retrosplenial cortex (RSCm), medial forebrain bundle (MBF), cornu ammonis field 2 (CA2). Data are mean ± SEM, n = 4–6. ^#^Significant increase versus cage controls p < 0.05 (Mann and Whitney test). (**b**) Endogenous albumin leakage expressed as the number of albumin spots after a cold injury. Upper graphs show representative images of albumin leakage identified as braun spots of albumin in cage control and positive control rats. Lower graph shows data of albumin leakage in the regions of interest ROI 1 (bregma –0.30 to −0.80 mm), ROI 2 (bregma −3.30 to −4.50 mm), and ROI 3 (bregma −7.30 to −8.00 mm) of cage-control and cold injury-treated rats. Data are mean ± SEM, n = 4–6. ^#^significant increase versus cage controls p < 0.05 (Mann and Whitney test).

After exposure to cold injury as a positive control (Fig. 1), a significant increase in degenerating neurons was detected using Fluoro-Jade B in brain slices from the regions located below the cold injury (motor cortex, retrosplenial cortex), whereas the other brain regions were less affected. Most animals (n = 6) showed distinct albumin leakage (spots) in the upper part of the brain, with clear albumin leakage around the unerlying vessels.

### Single RF exposure

A whole-brain analysis of degenerative neurons (Table 1) revealed an overall statistically significant decrease at 13 W/kg, 50 days after exposure to either GSM or UMTS signals. The number of degenerating neurons also significantly decreased at 0.26 W/kg, 7 days after a 2 h UMTS exposure.

**Table 1:** Brain-averaged numbers of degenerating neurons using Fluoro Jade B, following a single exposure (mean ± SD; n = 15–16). Data in italics correspond to statistically significant decreases compared to the sham group (p < 0.05).

SAR →	0 W/kg	0.026 W/kg		0.26 W/kg		2.6 W/kg		13 W/kg	
Time ↓	Sham	GSM	UMTS	*GSM*	UMTS	GSM	UMTS	GSM	UMTS
0 hour	0.40 ± 0.24	0.37 ± 0.13	0.34 ± 0.15	0.20 ± 0.13	0.23 ± 0.20	0.56 ± 0.36	0.47 ± 0.29	0.16 ± 0.16	0.14 ± 0.15
1 hour	0.34 ± 0.27	0.31 ± 0.22	0.37 ± 0.19	0.33 ± 0.23	0.29 ± 0.21	0.40 ± 0.35	0.46 ± 0.37	0.14 ± 0.12	0.28 ± 0.16
1 day	0.36 ± 0.32	0.26 ± 0.21	0.31 ± 0.27	0.39 ± 0.24	0.30 ± 0.14	0.33 ± 0.29	0.39 ± 0.30	0.20 ± 0.13	0.17 ± 0.16
7 days	0.36 ± 0.32	0.44 ± 0.14	0.44 ± 0.17	0.16 ± 0.16	*0.1 ± 0.1*	0.50 ± 0.31	0.40 ± 0.29	0.23 ± 0.16	0.21 ± 0.12
50 days	0.50 ± 0.33	0.43 ± 0.21	0.47 ± 0.33	0.45 ± 0.19	0.40 ± 0.20	0.39 ± 0.20	0.35 ± 0.26	*0.18 ± 0.14*	*0.10 ± 0.11*

Analysis of the data at the brain region level revealed that the number of degenerative neurons decreased in the medial forebrain bundle at 13 W/kg, immediately and 1 h after GSM exposure (Table S1a). Significant decreases in the number of degenerative neurons were found in several brain regions, versus their respective sham groups, at 7 and 50 days after exposure to UMTS at 0.26 and 13 W/kg. The most impacted brain areas were the amygdaloid nucleus and the dentate gyrus (Table S1b).

In terms of BBB permeation, no statistically significant variation was found between cage-control and sham-exposed animals at any time-point. At 13 W/kg, the extent of albumin extravasation was less than in sham samples for both RF signals. The effect was only found immediately and one day after exposure (Table 2).

**Table 2:** Endogenous albumin leakage following single exposure, expressed as the number of albumin spots (mean ± SD) in the three regions of interest: ROI 1 (bregma −0.30 to −0.80 mm), ROI 2 (bregma −3.30 to −4.50 mm), and ROI 3 (bregma −7.30 to −8.00 mm). Data in italics correspond to statistically significant decreases compared to the sham group (p < 0.05), n = 12–16.

Zone ↓	SAR →	0 W/kg	0.026 W/kg		0.26 W/kg		2.6 W/kg		13 W/kg	
	Time ↓	S	GSM	UMTS	GSM	UMTS	GSM	UMTS	GSM	UMTS
ROI 1	0 hour	2.13 ± 2.85	1.10 ± 1.17	1.35 ± 1.34	0.63 ± 0.96	1.03 ± 1.48	1.46 ± 1.29	1.47 ± 1.60	0.25 ± 0.41	*0.06 ± 0.25*
ROI 2	0 hour	2.84 ± 3.11	1.03 ± 0.64	1.35 ± 1.41	0.59 ± 0.66	0.91 ± 0.90	2.96 ± 3.37	2.25 ± 2.50	*0.19 ± 0.31*	*0.28 ± 0.48*
ROI 3	0 hour	2.88 ± 3.20	0.83 ± 1.01	2.12 ± 2.24	0.60 ± 0.71	0.72 ± 0.80	2.18 ± 2.16	1.34 ± 1.62	*0.00 ± 0.00*	*0.06 ± 0.25*
Brain (mean ± SD)	0 hour	2.61 ± 2.72	0.99 ± 0.63	1.60 ± 1.53	0.63 ± 0.58	0.87 ± 0.77	2.20 ± 2.08	1.69 ± 1.49	*0.15 ± 0.18*	*0.14 ± 0.27*
ROI 1	1 hour	0.84 ± 1.21	0.40 ± 0.47	0.56 ± 0.85	0.09 ± 0.20	0.75 ± 1.54	0.63 ± 0.74	0.87 ± 1.04	0.38 ± 0.62	0.09 ± 0.27
ROI 2	1 hour	1.03 ± 1.76	1.83 ± 1.84	1.19 ± 1.44	0.38 ± 0.47	1.19 ± 2.30	1.47 ± 1.89	1.10 ± 1.15	0.50 ± 0.52	0.31 ± 0.44
ROI 3	1 hour	1.19 ± 1.85	0.77 ± 0.75	1.19 ± 1.11	0.16 ± 0.30	0.97 ± 1.28	1.27 ± 1.44	1.10 ± 1.17	0.31 ± 0.40	0.25 ± 0.63
Brain (mean ± SD)	1 hour	1.02 ± 1.37	1.00 ± 0.75	0.98 ± 0.87	0.21 ± 0.23	0.97 ± 1.64	1.12 ± 0.90	1.02 ± 0.80	0.40 ± 0.38	0.22 ± 0.30
ROI 1	1 day	1.28 ± 1.02	0.53 ± 0.81	0.94 ± 1.21	1.41 ± 1.84	1.19 ± 1.09	1.07 ± 1.31	2.29 ± 2.71	0.59 ± 0.93	0.60 ± 1.27
ROI 2	1 day	1.47 ± 1.22	1.06 ± 0.95	1.97 ± 2.32	2.75 ± 3.32	2.22 ± 1.84	1.93 ± 2.73	3.21 ± 4.13	0.72 ± 0.71	0.53 ± 0.64
ROI 3	1 day	1.81 ± 1.41	0.88 ± 0.92	1.97 ± 2.06	2.97 ± 6.14	1.09 ± 0.99	2.67 ± 3.13	2.21 ± 3.38	0.53 ± 0.50	*0.38 ± 0.67*
Brain (mean ± SD)	1 day	1.52 ± 0.87	0.82 ± 0.73	1.63 ± 1.58	2.38 ± 3.49	1.50 ± 1.10	1.89 ± 2.07	2.57 ± 2.93	*0.61 ± 0.54*	*0.52 ± 0.66*
ROI 1	7 days	1.40 ± 1.38	0.81 ± 0.77	0.84 ± 1.27	1.64 ± 1.57	2.06 ± 2.62	1.43 ± 1.88	0.93 ± 1.10	1.44 ± 1.35	1.25 ± 1.24
ROI 2	7 days	2.17 ± 2.04	1.22 ± 1.24	0.91 ± 1.37	2.71 ± 2.04	1.66 ± 1.54	2.13 ± 1.64	1.67 ± 1.40	1.44 ± 1.14	1.63 ± 1.63
ROI 3	7 days	1.87 ± 1.49	1.66 ± 1.25	1.19 ± 0.95	1.11 ± 1.16	1.09 ± 1.20	2.70 ± 2.41	1.40 ± 1.76	0.94 ± 0.85	1.19 ± 1.34
Brain (mean ± SD)	7 days	1.81 ± 1.33	1.23 ± 0.86	0.98 ± 0.84	1.82 ± 1.47	1.60 ± 1.67	2.09 ± 1.49	1.33 ± 1.12	1.27 ± 0.84	1.35 ± 1.17
ROI 1	50 days	1.16 ± 1.04	1.40 ± 1.55	1.13 ± 0.85	1.50 ± 1.06	1.39 ± 1.78	0.93 ± 1.05	0.67 ± 1.16	0.53 ± 0.53	0.94 ± 0.75
ROI 2	50 days	2.44 ± 2.70	2.30 ± 1.11	2.59 ± 2.05	2.66 ± 2.13	1.77 ± 1.64	1.32 ± 1.25	1.20 ± 1.74	1.13 ± 0.65	1.28 ± 1.25
ROI 3	50 days	2.03 ± 2.36	3.50 ± 1.99	3.03 ± 2.04	2.66 ± 1.66	1.13 ± 0.95	1.25 ± 1.46	1.43 ± 1.91	1.09 ± 1.32	0.91 ± 0.71
Brain (mean ± SD)	50 days	1.88 ± 1.74	2.40 ± 1.02	2.25 ± 1.24	2.27 ± 1.19	1.50 ± 1.07	1.17 ± 1.05	1.10 ± 1.33	0.92 ± 0.67	1.04 ± 0.68

### Repeated exposures

**Figure 2:**
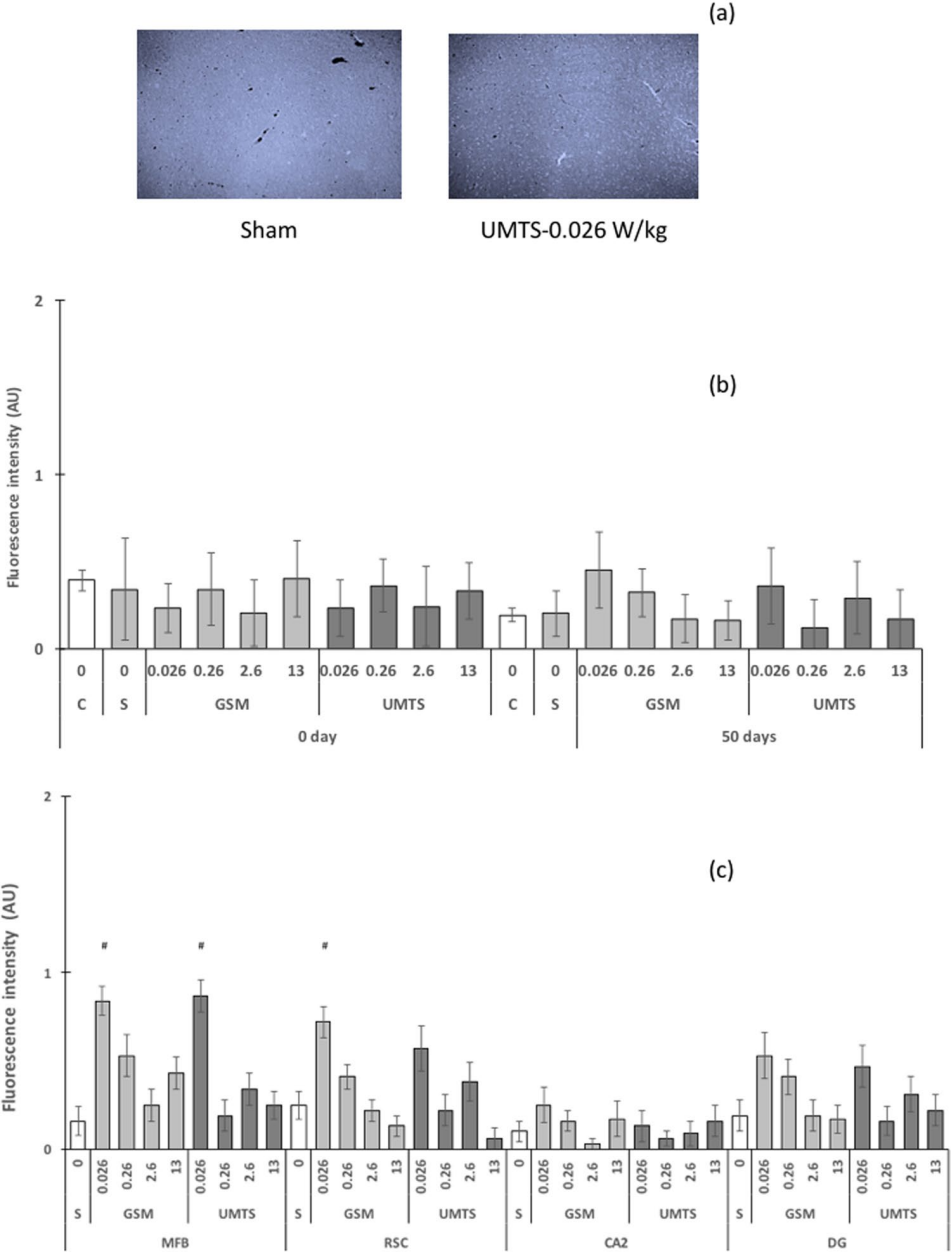
Degenerating neurons identified using Fluoro-Jade B 50 days after repeated exposures to GSM or UMTS. The rats were treated as indicated in the Methods. Brain-averaged SAR were 0 W/kg (C: cage controls, S: sham-exposed animals), 0.026 W/kg; 0.26 W/kg; 2.6 W/kg, and 13 W/kg. (**a**) Representative images of neuronal degeneration as detected using FluoroJade B in the medial forebrain bundle after sham-exposure or exposure to GSM-1800 at 0.026 W/kg. (**b**) Brain-averaged number of degenerating neurons at different BASAR levels, immediately (0 Day) or at 50 days. (**c**) Number of degenerating neurons in selected brain regions 50 days after repeated exposure to GSM-1800 and UMTS: medial forebrain bundle (MBF), medial retrosplenial cortex (RSC), cornu ammonis field 2 (CA2), and dentate gyrus (DG). Data are mean ± SEM; n = 13–16. ^#^significant increase versus sham exposure p < 0.05 (Kruskall-Wallis test).

Immediately after repeated UMTS or GSM exposure, no statistically significant changes in the number of degenerating neurons were found in the rat brains (Fig. 2b). Analysis of the data at the brain region level revealed that a single exposure significantly decreased the number of degenerating neurons in the posterior retrosplenial cortex after exposure to GSM at 2.6 W/kg (Table S2).

Fifty days after repeated GSM or UMTS exposure, no change in neuron degeneration was detected at whole-brain level (Fig. 2b). However, at a BASAR of 0.026 W/kg, a statistically significant increase in degenerating neurons was detected in the medial retrosplenial cortex and the medial forebrain bundle following GSM exposure and in the medial forebrain bundle following UMTS exposure (Fig. 2a,c, Table S2).

**Figure 3:**
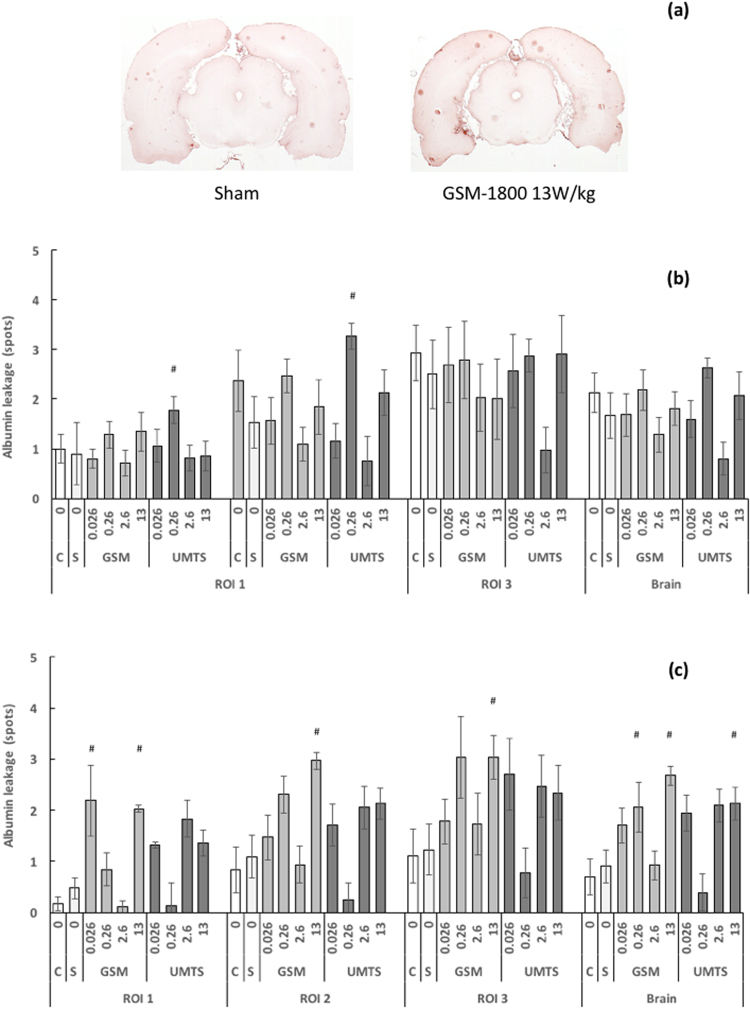
Endogenous albumin leakage expressed as the number of albumin spots following repeated exposure to GSM-1800 or UMTS-1950: The rats were treated as indicated in the Methods. Brain-averaged SAR were 0 W/kg (C: cage controls, S: sham-exposed animals), 0.026 W/kg; 0.26 W/kg; 2.6 W/kg, and 13 W/kg and the regions of interest were: ROI 1 (bregma −0.30 to −0.80 mm), ROI 2 (bregma −3.30 to −4.50 mm), and ROI 3 (bregma −7.30 to −8.00 mm). (**a**) Representative images of endogenous albumin leakage after sham-exposure or exposure to GSM-1800 at 13 W/kg in the third region of interest (bregma −7.30 to −8.00 mm). (**b**) Endogenous albumin leakage immediately after a 4-week exposure. (**c**) Endogenous albumin leakage 50 days after a 4-week exposure. Data are mean ± SEM; n = 11–16. ^#^Significant increase versus sham exposure, p < 0.05 (Kruskall-Wallis test).

There was no significant difference in albumin extravasation, detected as albumin “spots” in the brain, between cage controls and sham-exposed animals at any time-point. Fifty days after repeated GSM exposure, increased albumin extravasation was observed in the first brain region of interest only at 0.026 W/kg, in the whole brain at 0.26 W/kg, and in all regions of interest at 13 W/kg, with values ranging from 2.03–3.03, compared to 0.47–1.23 in sham-exposed brains (Fig. 3a,b).

UMTS increased albumin extravasation immediately after exposure at 0.26 W/kg (ROI 1 and ROI 2, Fig. 3b) but the effect was reversible at 50 days. At 13 W/kg, the increase in the brain-averaged number of albumin spots was statistically significant 50 days after exposure (Fig. 3c).

Overall, the magnitude of the RF effect was not very large. This is exemplified by the fact that the largest number of albumin spots observed following GSM repeated exposure at 13 W/kg was 3.0 ± 0.6 in the third brain region of interest at 50 days, i.e. similar to the maximum observed in cage-control rats (2.9 ± 0.6 spots, Fig. 3). In the same brain region, the positive-control group exhibited 13.8 ± 2.8 spots, versus 4.83 ± 1.64 spots in the corresponding cage-control group (Fig. 1b).

## Discussion

Neuronal degeneration following exposure to mobile phone-related RF had previously been investigated by a single research group^11,12^. In these papers, the authors reported the presence of so-called “dark neurons” 50 days after a single 2-hour exposure of rats to GSM-900. Rats were whole-body exposed at 0.002, 0.02, and 0.20 W/kg, and a SAR-dependent increase in the occurrence of dark neurons was detected using Cresyl violet. Salford *et al*. (2003) made the assumption that dark neurons were degenerative. Cresyl violet dye is a conventional Nissl stain for cytoplasm, staining both neurons and glia in brain slices. The specific feature of dark neurons, however, occurs after ischemia and may be defined as very strongly-stained, shrunken cells, with no distinguishable nucleus. This feature is not specific to neuronal degeneration; it may be reversible and artefacts during tissue handling may lead to false positives^31,32^.

Therefore, in this study, we used Fluoro-Jade B, since Fluoro-jade derivatives are considered to be reliable markers of degenerating neurons^33,34^. As previously published concerning GSM-900^17^, the present data, from a study extended to two other RF signals, i.e. GSM-1800 and UMTS, did not reveal any detrimental impact of acute exposure to these signals on neuronal degeneration. Our data even indicated that fewer degenerating neurons were detected following a single 2-hour RF exposure than in the sham-exposed group. The biological relevance of this effect is not clear, but similar data were found for albumin extravasation.

Repeated GSM or UMTS exposure at all BASAR levels did not alter the number of degenerating neurons in the rat brain, compared to sham-exposed rats, with the exception of the lowest exposure level in 2 out of the 12 brain regions considered in this study: at a BASAR of 0.026 W/kg, the retrosplenial cortex and medial forebrain bundle locally experienced SARs of 0.047 and 0.024 W/kg, respectively (Table 3). At those levels, the temperature increase was negligible. As no statistically significant increase in degenerating neurons was detected in brain regions exposed at similar and higher levels, this was probably a chance finding.

**Table 3:** SAR values in selected regions for the four BASAR exposure levels at 1800 MHz.

SELECTED REGION	BASAR − 1800 MHz			
	13 W/kg	2.6 W/kg	0.26 W/kg	0.026 W/kg
	Selected region SAR (mean ± SD, W/kg)			
Motor cortex (MC)	22.7 ± 0.2	4.55 ± 0.04	0.45 ± 0.01	0.045 ± 0.001
Medial forebrain bundle (MFB)	12.2 ± 1.5	2.44 ± 0.30	0.24 ± 0.03	0.024 ± 0.003
Retrosplenial cortex (RSC, median)	23.3 ± 0.2	4.67 ± 0.04	0.47 ± 0.01	0.047 ± 0.001
Auditory cortex (Aud)	12.6 ± 0.6	2.51 ± 0.12	0.25 ± 0.01	0.025 ± 0.001
Amygdaloid nucleus (Amyg)	7.5 ± 0.6	1.50 ± 0.12	0.15 ± 0.01	0.015 ± 0.001
Dentate Gyrus (DG)	18.8 ± 0.4	3.77 ± 0.07	0.38 ± 0.01	0.038 ± 0.001
Cornu Ammonis field 1 (CA1)	21.0 ± 1.0	4.21 ± 0.19	0.42 ± 0.02	0.042 ± 0.002
Cornu Ammonis field 2 (CA2)	19.4 ± 0.1	3.87 ± 0.02	0.39 ± 0.01	0.039 ± 0.001
Cornu Ammonis field 3 (CA3)	17.7 ± 0.3	3.54 ± 0.05	0.35 ± 0.01	0.035 ± 0.001
Dorsomedial periaqueductal gray (dmPAG)	15.8 ± 0.4	3.17 ± 0.07	0.32 ± 0.01	0.032 ± 0.001
Retrosplenial cortex (RSC, posterior)	21.4 ± 3.6	4.28 ± 0.73	0.43 ± 0.07	0.043 ± 0.007
Pontine nuclei (PN)	7.8 ± 1.7	1.56 ± 0.34	0.16 ± 0.03	0.016 ± 0.003

In summary, single or repeated 2-hour exposures to GSM-1800 and UMTS had a negligible effect on degeneration in the rat brain.

These results indicate no alteration in BBB permeability after acute exposure to mobile phone-RF signals up to 13 W/kg and up to 50 days after exposure. The data obtained immediately after repeated exposure at BASAR levels up to 13 W/kg were generally negative, in agreement with previously-published data.

The Salford group reported that, when rats were exposed to GSM, 2 h per week for 55 weeks, at whole-body SAR ranging from 0.06 to 0.6 W/kg, no albumin extravasation, dark neurons, lipofuscin aggregation, or signs of cytoskeletal or neuronal changes were observed^35^. Moreover, after 5 weeks' exposure (900 MHz mobile phone signal for 2 h/day, 5 days/week) at whole-body average SAR of 0.3 or 3.0 W/kg, no degenerative changes, dying neurons, or effects on blood-brain barrier leakage were detected in young male Wistar rats^36^. The Finnie group published a series of reports on the effects of RF on the BBB in mice^19,20,23^. In all these studies, whole-body GSM-900 exposure, mainly at 4 W/kg, with varying exposure times and durations, the findings were as follows: (i) 60-minute daily exposure, 5 days per week, for 104 weeks at 0.25, 1.0, 2.0, and 4.0 W/kg produced negligible disruption to BBB integrity (2002); (ii) no albumin extravasation was found in control mice or those exposed for 60 min/day from day 1 to day 19 of gestation (2006); (iii) following daily exposure 5 days per week for 104 weeks, no up-regulation of the water channel protein AQP-4 was detected, suggesting that there was no significant increase in BBB permeability (2009).

National and European reviews, based on a thorough analysis of the literature on BBB studies in rodents exposed to mobile telephony RF, have concluded that BBB permeability is not affected by acute or chronic RF exposure, irrespective of the signal used, within the following ranges: 898–24500 MHz, whole-body SAR 0.25–4 W/kg, and BASAR 0.3–6 W/kg^30^. Hence, our results are in agreement with that conclusion, with the exception of the very high SAR, 13 W/kg, used in these experiments.

In the present work, BBB permeability in the whole rat brain increased significantly 50 days after repeated exposures: 3-fold for GSM and 2.4-fold for UMTS at 13 W/kg. A similar significant effect was seen in the whole brain with GSM-1800 at 0.26 W/kg. However, while the mean number of spots was quite similar at different BASAR levels, their distribution among the animals varied. For example, 20% of the 0.026 W/kg rats had between 4 and 5.5 spots, versus 45% of the 13 W/kg rats (data not shown). Thus, the effect was much stronger and consistent at 13 W/kg than at 0.026 W/kg or 0.26 W/kg. It is also noteworthy that the highest albumin levels were comparable to the highest background levels in cage-control rats (Fig. 3).

Therefore, our overall conclusion is that physiopathological consequences are unlikely to occur in the rat brain up to 50 days after single or immediately after repeated exposure to GSM or UMTS, at BASAR up to 13 W/kg. The delayed deterioration in BBB integrity after repeated exposures at 13 W/kg is probably due to temperature elevation in the brain (ca. 0.9 °C, Figure S1). The role of temperature elevation in biological tissues, whether or not it is caused by RF exposure, is a well-established source of detrimental biological and health effects^37^. One example relevant to our study is that RF exposure is known to increase blood flow in the brain together with temperature in the cortex^38^. It is thus most likely that effects observed at low BASAR in some brain regions, at very low temperature elevations, are chance findings and not actual RF effects.

In this work, the delayed effect may have been caused by repeated temperature elevations in the brain, gradually inducing an alteration in the BBB permeability. The underlying mechanisms will need to be investigated further.

To put this BASAR of 13 W/kg into perspective, the maximum SAR in the periphery of the rat brain was ca. 26 W/kg, i.e. twice the BASAR where the effect was found^39^. According to Ammari *et al*.^40^, extrapolation to the human situation gives an SAR_10g_ of ca. 50 W/kg, i.e. much higher than the 2 W/kg SAR_10g_ exposure limit, but half the 100 W/kg level critical effect, as defined by ICNIRP. Whether local exposure of the brain to RF may be differentiated from local exposure of other parts of the body is thus an open question.

## Methods

### Animals

Six-week-old (200–225 g for single exposures) and 10-week-old (300–325 g for repeated exposures) male Wistar-Han rats (Janvier, Le Genest Saint Isle, France) were housed under controlled temperature (22 ± 1 °C) and lighting conditions (monitored light-dark cycles 08:00–20:00), and supplied with water and UAR 04 food (Safe, Augy, France) *ad libitum*. Animals were kept for one week in the facility before starting the experimental procedure. Cages were cleaned twice a week. All French national regulations were implemented in housing and handling the animals.

During the experiment, animals were weighed at least once per week to monitor their physiological development.

All experimental procedures were carried out in compliance with the CNRS ethical rules and the European Community Council Directive for the Care and Use of laboratory animals (2010/63/EU). Protocols were approved by the institutional ethics committee (Comité d’éthique pour l’Expérimentation Animale Bordeaux, approval number 00237.01).

### Radiofrequency exposure setup

**Figure 4:**
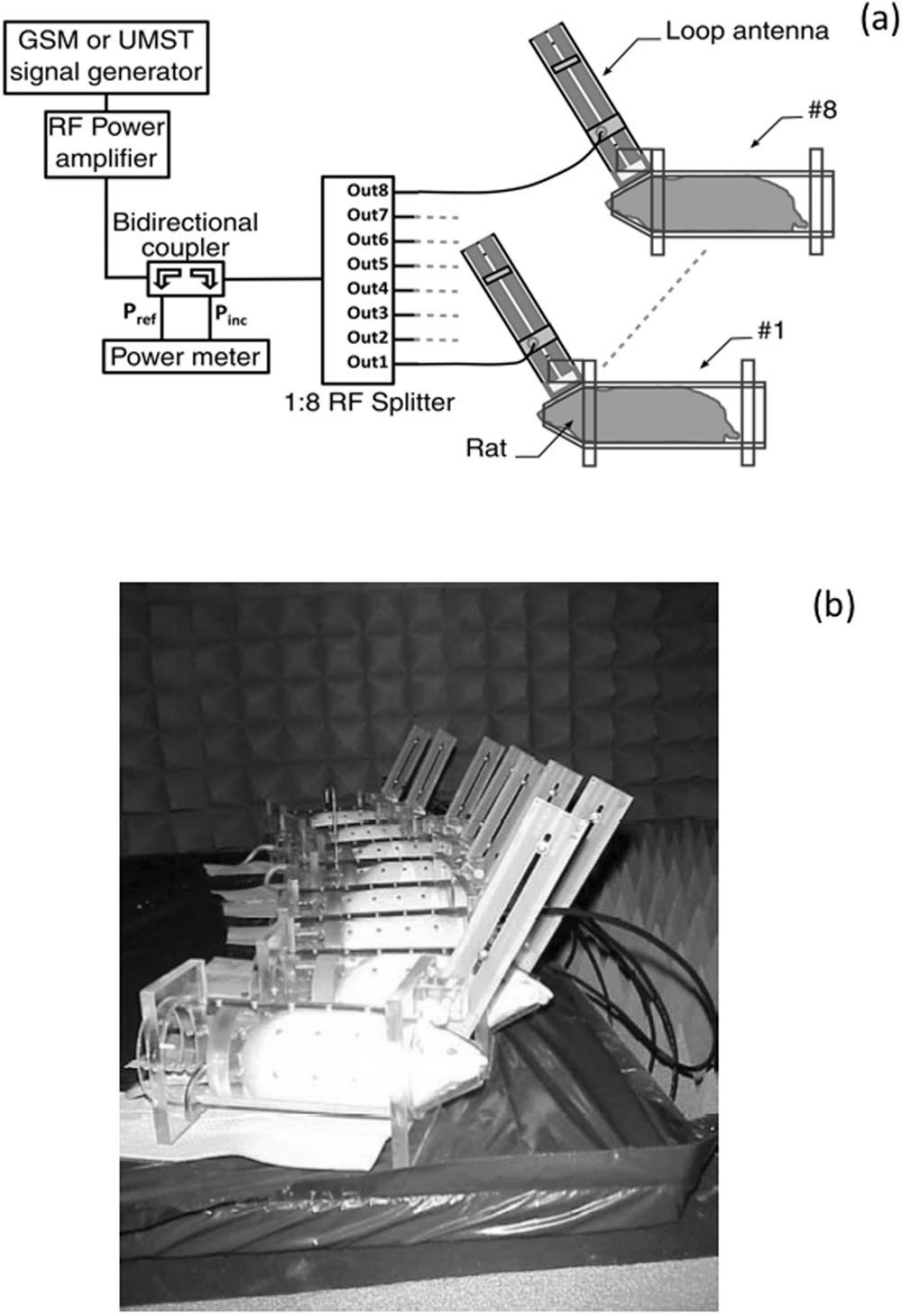
Loop exposure setup. The setup is designed for head-only exposure. Antennas were adapted at either 1800 or 1960 MHz. (**a**) Diagram of the exposure setup for rat exposure at 1800 and 1960 MHz. (**b**) Photograph of the exposed animals, placed in rockets fitted with antennas.

The signals used in this study were GSM-1800 MHz and UMTS-1960 MHz. At 1800 MHz, the GSM field applied was an amplitude-modulated signal with rectangular pulses at a repetition frequency of 217 Hz and a duty cycle of 1:8, yielding a frame length of 4.61 ms, each including a 576 µs burst. The UMTS signal was produced by a UMTS generator (GUS 6960S, University of Wuppertal, Wuppertal, Germany)^41^ coupled to an RF power amplifier (14002600-10, RFPA S.A., Artigues-près-Bordeaux, France). Head-only exposure was achieved using a loop antenna (Fig. 4).

The SAR in the brain, or BASAR, had previously been characterized at 900 MHz^39^ and 1800 MHz^42,43^. Temperature was measured inside a homogeneous gel phantom using a thermistor probe (VITEK, BSD Medical Devices, Salt Lake City, USA), 1.3 cm from the inner side of the rocket, directly below the loop. The SARs obtained from these measurements were compared with numerical simulations based on the Finite Difference Time Domain (FDTD) method. This very powerful, popular tool in bioelectromagnetics was also applied using a 7-tissue numerical rat phantom. For GSM and UMTS signals, the BASAR was 8.4 ± 0.3 and 7.9 ± 0.3 W/kg/W, at 1800 MHz and 1960 MHz, respectively.

If the BASAR is considered to be the “local” SAR level, with a 2-fold range of voxel SAR levels, then the levels in this set of experiments were 0.026 (very low), 0.26 (low), 2.6 (in the range of the ICNIRP limits for local public exposure), and 13 W/kg (in the range of ICNIRP limits for local occupational exposure).

*In vivo* temperatures were measured on adult, conscious, exposed rats by inserting temperature probes next to the dura matter and into the rectum. Temperature elevation in the cortex (with respect to temperature change in the rectum) was 0.9 °C at 13 W/kg (Figure S1). We noted that, immediately after the end of exposure, the relative temperature dropped very rapidly and went almost back to background levels within 5 minutes. Therefore, rats were experiencing repeatedly temperature increases in their brains during 2 hours/day, 5 days/week for 4 weeks at BASAR of 13 W/kg.

**Figure 5:**
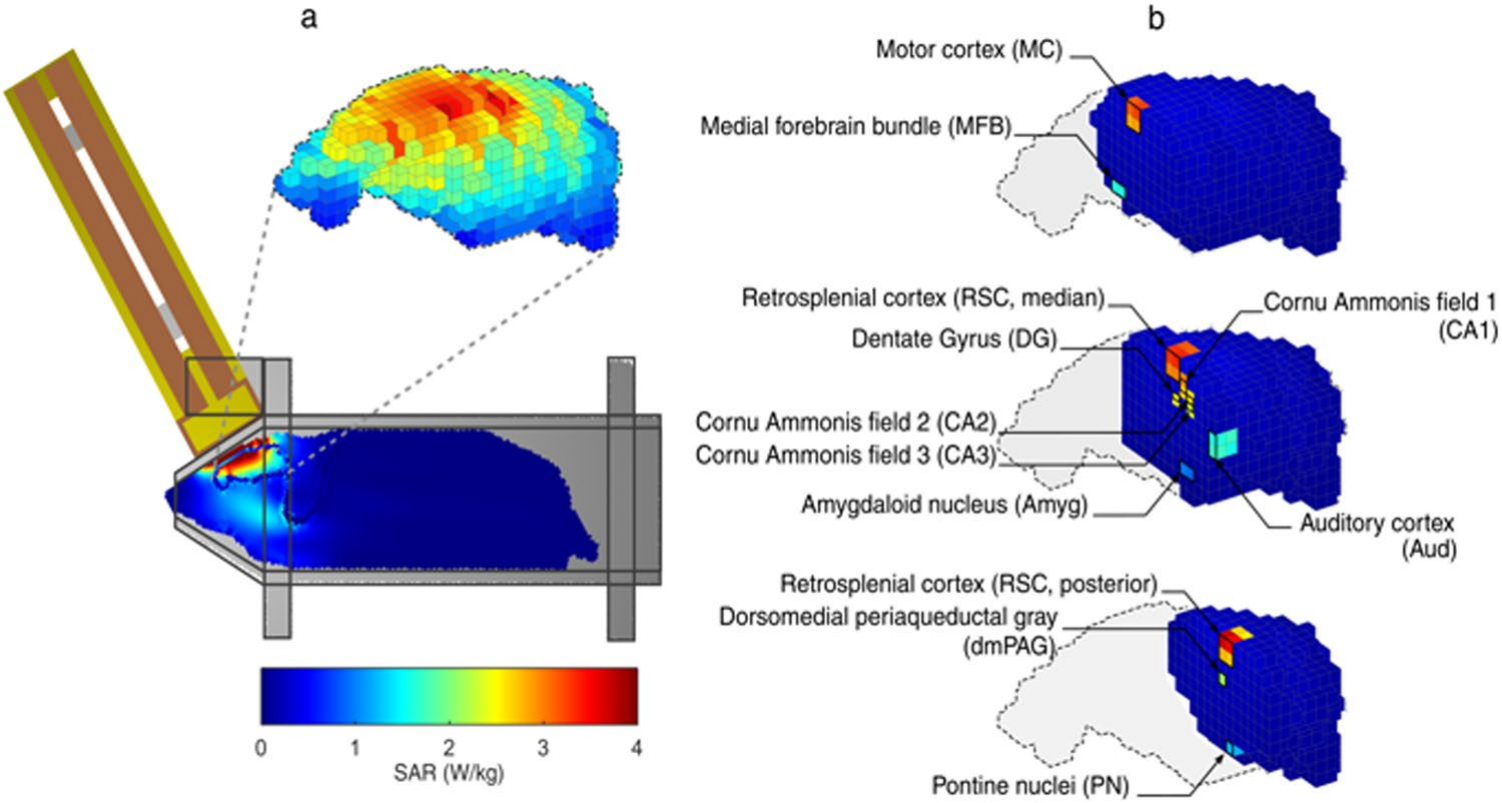
Dosimetric analysis of SAR in the rat brain during exposure to GSM 1800 MHz. A heterogeneous rat model was used to evaluate local SAR in the selected regions. (**a**) Global view of the rat phantom in the rocket with the loop antenna matched at 1800 MHz and 3D view of brain SAR distribution. (**b**) SAR corresponding to the selected regions.

SAR values were computed for the whole head and in the regions selected for biological analysis. The SAR spatial distribution (Fig. 5a) illustrates the distribution of exposure, with the highest SAR values in the brain close to the loop antenna. The SAR values in the selected areas at 1800 MHz are shown in Fig. 5b and reported in Table 3. Similar results were observed at 1960 MHz. Comparable exposure systems and dosimetric analyses^43^, with assessment of the stability of the exposure system^39^, had been previously reported.

### RF Exposure protocols

#### Single exposure to radiofrequency fields

Rats were divided into 6 groups of 8: control-cage group, sham-exposed group, and 4 groups exposed to the GSM or UMTS mobile telephony-related signals. Sham-exposed rats (restrained in a rocket), cage controls, and positive controls (cold-shock) were included in the protocol. After a one-week acclimatization period, exposed groups were gradually trained to the rocket-type exposure over one week (0.5, 1, 1.5, and 2 h/day over 5 days). Consequently, rats were enrolled in the single 2-h exposure protocol at an age of 12 weeks. A series of experiments were conducted according to the time elapsed after exposure (0 and 1 h, 1, 7, and 50 days), and two independent series were performed for each exposure condition, with a total of 16 animals per group.

#### Repeated exposure to radiofrequency fields

After a one-week acclimatization period and one week of gradual training in the rocket-type exposure setup, as described above, rats were exposed repeatedly (2 h/day, 5 days/week, 4 weeks). Sham-exposed rats (restrained in a rocket), cage controls, and positive controls (cold-injury) were included in the protocol. The tests were performed immediately and 50 days after exposure. Eight independent series were performed for each exposure condition.

### Biological samples

Rats were euthanized using isoflurane inhalation (TEM, Bordeaux, France), followed by transcardiac perfusion with phosphate buffer saline (PBS) containing heparin (5 U/ml) for 8 minutes, and fixed with 4% Paraformaldehyde in 0.1 M phosphate buffer for 8 minutes (Sigma Aldrich, Saint Quentin Fallavier, France). The brains were removed and kept in the fixative solution at 4 °C overnight. They were dehydrated using 20% sucrose in phosphate buffer at 4 °C for 48 h and then cryo-preserved using isopentane at −80 °C. To ensure blinding of the experiments, brains were coded at this stage, before slicing and analysis.

Serial 10-µm brain sections were prepared from 3 regions of interest: ROI 1 (bregma −0.30 to −0.80 mm), ROI 2 (bregma −3.30 to −4.50 mm), and ROI 3 (bregma −7.30 to −8.00 mm). A total of 12 regions in the cortex and hippocampus were then examined in each slice: the motor cortex (MC) and medial forebrain bundle (MFB) in the frontal zone, the retrosplenial cortex (RSC), auditory cortex (Aud), amygdaloid nucleus (Amyg), cornu ammonis field 1 (CA1), cornu ammonis field 2 (CA2), cornu ammonis field 3 (CA3), and dentate gyrus (DG) in the median zone, and the dorsomedial periaqueductal gray (dmPAG), retrosplenial cortex (RSC), and pontine nuclei (PN) in the posterior zone.

Two slices were analyzed per region of interest and the average value was taken into account for statistical analysis. The results are expressed as the mean ± SD of a given group. The mean of the three regions of interest was then considered representative of the degenerating neuronal population in the whole brain.

#### Detecting degenerative neurons

Fluoro-Jade B staining was performed on tissue slices treated with successive baths: (i) 1% NaOH and 80 % ethanol for 5 min, (ii) 70% ethanol for 2 min, and (iii) 0.06% potassium permanganate for 10 min. They were rinsed and stained using a 0.001% Fluoro-jade B solution (30 min, gentle agitation). Slices were rinsed in distilled water and immersed in xylene. Cover slips were mounted on slides for microscopy observation (Axiovert 40 C Zeiss, Germany) and visual analysis. Two slices from each region of interest were examined per condition and one x100 representative microscopic photograph was taken. On these photographs, degenerating neurons were identified as green fluorescent cells. Fluorescence intensity was scored on the following scale: 0 for no labelling, 1 for medium labelling, and 2 for strong labelling.

#### Detection of endogenous albumin

Permeation of the BBB was assessed by the presence of endogenous albumin in the brain tissue. Briefly, endogenous peroxidase was quenched with 0.3% H_2_O_2_ in 0.3% horse serum (HS) in PBS. Non-specific binding sites were saturated with PBS-HS (5%, 10 min). Tissue sections were incubated with an anti-human albumin antibody (Dakocytomation, France) diluted in PBS/HS (1/2000) for 1 h and the coupling of the antibody revealed using an indirect immunoperoxidase method (Vectastain ABC kit, Vector SA, France). Cover slips were mounted on slides before microscopy observation and whole brain sections were analyzed using visual quantification.

Endogenous albumin leakages, which appeared as brown spots around the vessels, were carefully identified to exclude staining artefacts and counted on two slices per condition.

#### Positive controls

Cold injury was used to induce brain damage as a positive control treatment. A total of 6 rats were subjected to cold shock. After anesthesia (Isoflurane), the head skin was incised in the parietal region and the skull surface exposed. A small block of dry ice was maintained on one side of the hemisphere for 5 minutes. Then the rat was left without treatment and kept warm for 25 minutes, and then anesthetized for fixation and brain removal.

### Statistical analysis

Statistical analysis was performed using non-parametric tests, in collaboration with a biostatistician (Prof. Le Pape, University of Tours, France), using Statview software (SAS Institute Inc., Cary, NC, USA). Non-parametric tests were chosen as the number of experimental animals never exceeded 16, so the ANOVA tests were not appropriate. The Kruskall-Wallis test with correction for multiple comparisons (Dunn test) was used for the various experimental conditions (sham-exposed and 8 BASARs: 4 GSM and 4 UMTS). Each brain region was analyzed separately at each time-point after exposure. When a significant p value (<0.05) was found, a Mann-Whitney test was performed on the sham and exposed groups (all BASARs). Cage controls and positive controls were also compared using the Mann-Whitney test. For all tests, a p value <0.05 was considered significant. Based on historical controls, the use of 16 rats per group was adequate for detecting 50 and 37% (effect size) increases in albumin and Fluorojade B, respectively, with a statistical power of 90% (one tail, alpha = 0.05).

## Electronic supplementary material

Supplementary Dataset 1
