# Supplementary material for: Effects of GSM and UMTS mobile telephony signals on neuron degeneration and blood-brain barrier permeation in the rat brain
Source: Sci Rep. 2017 Nov 14;7:15496. doi: 10.1038/s41598-017-15690-1 (PMC5686211; doi:10.1038/s41598-017-15690-1)
Supplement: Supplementary file 1 — Supplementary Dataset 1 [file 41598_2017_15690_MOESM1_ESM.doc]

# Effects of GSM and UMTS mobile telephony signals on degenerating neurons and blood-brain barrier permeation in the rat brain

Florence Poulletier de Gannes^1*^, Hiroshi Masuda^2*^, Bernard Billaudel^1^, Emmanuelle Poque-Haro^1^, Annabelle Hurtier^1^, Philippe Lévêque^3^, Gilles Ruffié^1^, Murielle Taxile^1^, Bernard Veyret ^1,4^, and Isabelle Lagroye^1,4+^

^1^University of Bordeaux, IMS laboratory UMR-5218 CNRS, Talence, F-33405, France

^2^ Kurume University School of Medicine, Department of Environmental Medicine, Kurume, Fukuoka J-830-0011, Japan

^3^ University of Limoges, CNRS, XLIM, UMR 7252, Limoges, F-87000, France

^4^ Paris Sciences et Lettres Research University, Paris, F-75005, France

**Supplementary data**

**Legends**

**Supplementary Table S1a:** **Degenerating neurons using Fluoro-Jade following a single 2-h GSM exposure**. Data of degenerating neurons, identified using Fluoro-Jade B in cage-control (C), and GSM exposed rats versus sham-exposed (S) rats (mean ± SEM). Four BASARs and 12 brain regions were tested: motor cortex (MC), medial forebrain bundle (MFB), retrosplenial cortex (RSC, median and posterior), auditory cortex (Aud), amygdaloid nucleus (Amyg), cornu ammonis field 1 (CA1), cornu ammonis field 2 (CA2), cornu ammonis field 3 (CA3), dentate gyrus (DG), dorsomedial periaqueductal gray (dmPAG), pontine nuclei (PN). Averaged brain values are also given. Numbers in italics and bold correspond to statistically significant differences compared to the sham group (+ for increases and – for decreases). Numbers of animals per group are under parentheses.

**Supplementary Table S1b: Degenerating neurons using Fluoro-Jade following a single 2-h UMTS exposure**. Data of degenerating neurons, identified using Fluoro-Jade B in cage-control (C), and GSM exposed rats versus sham-exposed (S) rats (mean ± SEM). Four BASARs and 12 brain regions were tested: motor cortex (MC), medial forebrain bundle (MFB), retrosplenial cortex (RSC, median and posterior), auditory cortex (Aud), amygdaloid nucleus (Amyg), cornu ammonis field 1 (CA1), cornu ammonis field 2 (CA2), cornu ammonis field 3 (CA3), dentate gyrus (DG), dorsomedial periaqueductal gray (dmPAG), pontine nuclei (PN). Averaged brain values are also given. Highlighted numbers correspond to statistically significant differences compared to the sham group (yellow for increases and green for decreases). Numbers of animals per group are under parentheses.

**Supplementary Table S2: Degenerating neurons as identified using Fluoro-Jade B after repeated exposures to GSM and UMTS**. Kruskall Wallis test was used on data on degenerating neurons as identified using Fluoro-Jade B in cage-control, and GSM-1800- or UMTS-exposed rats versus sham-exposed rats (mean ± SEM). Different SAR levels and different brain regions were tested: motor cortex (MC), medial forebrain bundle (MFB), retrosplenial cortex (RSC, median and posterior), auditory cortex (Aud), amygdaloid nucleus (Amyg), cornu ammonis field 1 (CA1), cornu ammonis field 2 (CA2), cornu ammonis field 3 (CA3), dentate gyrus (DG), dorsomedial periaqueductal gray (dmPAG), pontine nuclei (PN). For each parameter studied, statistical analysis was performed between sham-exposure and exposure to GSM-1800- or UMTS (all SAR levels) for each brain zone and time after exposure. A p value < 0.05 versus sham-exposed rats (S) was considered as significant. Highlighted numbers correspond to statistically significant differences compared to the sham group (yellow for increases and green for decreases). Numbers of animals per group are under parentheses.

**Supplementary Figure S1 : Temperature measurement in the rat brain during a 2-hour RF exposure.** Heads of conscious rats were placed just under a loop antenna and locally exposed to RF for 2 hours (0-120 min).

(a) Temperatures in cortex and rectum were simultaneously measured before, during, and after UMTS exposure at 0, 5, and 15 W/kg of BASAR. ΔTc-r (in °C) is the relative value of temperature change in cortex with respect to temperature change in rectum. The black line is the mean value and grey bar is SEM (n=3). ΔTc-r was +0±0.1°C at 0 W/kg (sham-exposure), +0.4±0.1°C at 5 W/kg, and +1.0±0.0°C at 15 W/kg. (b) Example of temperature change in cortex with respect to temperature change in rectum of rats using BASAR of 13 W/kg as used in this work (n=1). Temperature was measured during exposure to GSM-1800 or UMTS signal for 2 hours (as during the experimental procedure). ΔTc-r was then calculated to be +0.9°C .

**Table S1a**

**Table S1b**


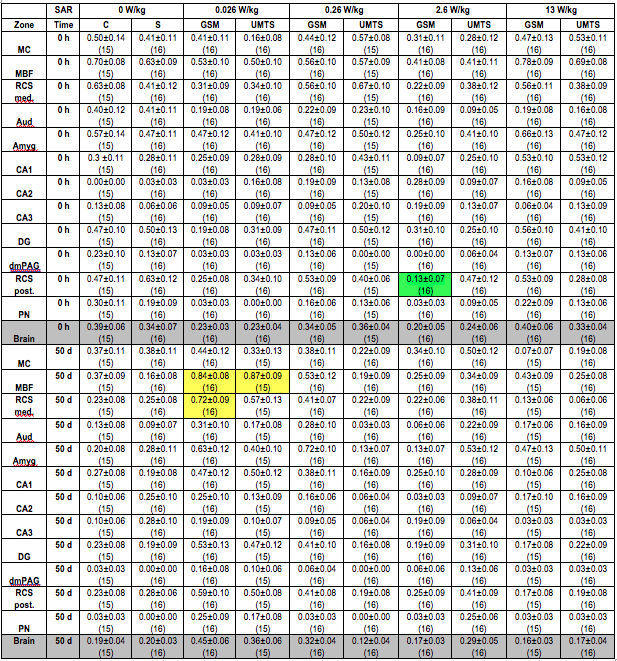


**Table S2**

(a)

*
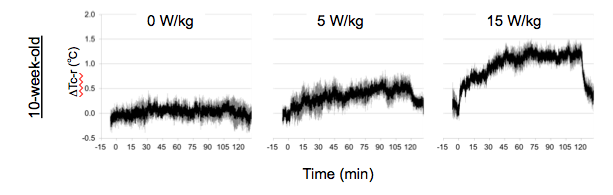
*

(b)

**Figure S1**
